# Postgraduate psychological stress detection from social media using BERT-Fused model

**DOI:** 10.1371/journal.pone.0312264

**Published:** 2024-10-31

**Authors:** Muni Zhuang, Dongsheng Cheng, Xin Lu, Xu Tan

**Affiliations:** 1 National Institute for Data Science in Health and Medicine, Xiamen University, Xiamen, Fujian, China; 2 School of Medicine, Xiamen University, Xiamen, Fujian, China; 3 Shenzhen Institute of Information Technology, School of Software Engineering, Shenzhen, Guangdong, China; 4 College of Systems Engineering, National University of Defense Technology, Changsha, Hunan, China; 5 Shenzhen Institute of Information Technology, Career-Oriented Multidisciplinary Education Center, Shenzhen, Guangdong, China; Air Force Engineering University, CHINA

## Abstract

Postgraduate students face various academic, personal, and social stressors that increase their risk of anxiety, depression, and suicide. Identifying cost-effective methods of detecting and intervening before stress turns into severe problems is crucial. However, existing stress detection methods typically rely on psychological scales or devices, which can be complex and expensive. Therefore, we propose a BERT-fused model for rapidly and automatically detecting postgraduate students’ psychological stress via social media. First, we construct an improved BERT-LDA feature extraction algorithm to extract group stress features from large-scale and complex social media data. Then, we integrate the BiLSTM-CRF named entity recognition model to construct a multi-dimensional psychological stress profile and analyze the fine-grained feature representation under the fusion of multi-dimensional features. Experimental results demonstrate that the proposed model outperforms traditional models such as BiLSTM, achieving an accuracy of 92.55%, a recall of 93.47%, and an F1-score of 92.18%, with F1-scores exceeding 89% for all three types of entities. This research provides both theoretical and practical foundations for universities or institutions to conduct fine-grained perception and intervention for postgraduate students’ psychological stress.

## 1. Introduction

### 1.1 Motivation

Postgraduate education stands as the pinnacle of the educational pyramid, serving as a crucial catalyst for national economic and social advancement. Globally, the number of postgraduate students has significantly increased over the past two decades. Particularly in China, the number of postgraduate students skyrocketed from 629 in 1949 to 3.883 million in 2023, representing a 6000-fold increase. However, owing to factors such as enrollment scale and socio-environmental context, mental health concerns have become increasingly common among postgraduate students. In contrast to physical illnesses, mental health problems tend to be overlooked and untreated, especially among postgraduate students who may lack awareness of mental health or be reluctant to disclose their challenges. This has led to a concerning increase in suicide rates among postgraduate students. The COVID-19 pandemic and other major public crises have further impacted the psychological stress of postgraduates. These events have disrupted their daily lives (e.g., mandatory isolation, online teaching, and assessments), exacerbating emotions such as fear, frustration, isolation, anxiety, and depression, and leading to an increase suicidal ideation [1–3].

Stress refers to individuals’ emotional responses, such as fear, anger, and anxiety towards their environment [4]. Numerous studies have demonstrated that excessive stress is a significant risk factor for mental health problems [5]. Currently, research on postgraduate stress primarily emphasizes qualitative analysis. According to Chen, the primary sources of stress for postgraduates include research field, thesis work, career prospects, financial concerns, physical appearance, interpersonal relationships, living environment, emotional well-being, and learning [6]. Levecque et al. suggested that the sources of stress for doctoral students include university policies, work-family balance, work requirements, supervisors’ guidance style, team decision-making culture, and career aspirations beyond academia [7]. Harvard University conducted an evaluation of stress among finance doctoral students at eight American universities and found that significant stressors include study time, research experience, peer pressure, social support, financial stress, and the relationship with the supervisors [8]. Liu studied stress among doctoral students in medical schools and found that academic performance, frequency of meetings with supervisors, difficulty in publishing papers, work-family balance, self-efficacy, and the guidance relationship are the main factors [9].

The assessment of stress and mental health typically relies on clinical data (e.g., brain MRI, EEG, interview records, rating scales) or sensor data from smart and wearable devices [10]. However, these diagnosing methods are time-consuming and may lack spontaneity. With the widespread use of mobile internet and social media (including Twitter, Reddit, Microblogs, etc.), postgraduates are more inclined to express their stress and emotions online. Those experiencing high levels of stress often post about depression and therapy on social media, indicating that social media content contains valuable information for identifying depressive tendencies [11]. Utilizing social media for low-cost, non-clinical predictive stress assessment and diagnosis can enable timely prevention of self-harm and suicide attempts [12]. Furthermore, large-scale social media data provide researchers with the opportunity to quickly understand the stress levels and mental health status of postgraduates [13]. For example, Liang et al. evaluated the stress of over 60,000 college students in China through Weibo and found that 33.8% of them had high stress levels [14]. Saha et al. assessed the psychological stress of American college students who experienced 12 campus shooting incidents over five years using Reddit. The study found that the stress resulting from such incidents differed from typical ones [15].

In recent years, the combination of computational linguistics and clinical psychology to detect public psychological stress has become a research hotspot. Traditional machine learning can quickly, automatically, and objectively assess public psychological stress, but their performance largely depends on feature construction and selection. Due to the limitations of features and algorithms, their generalization ability is often weak. In contrast, deep learning can understand the contextual semantics of complex natural language sentences, fundamentally transforming the feature extraction process used in traditional machine learning. Existing deep learning-based stress detection algorithms can perform preprocessing, feature extraction, and stress recognition in a continuous process, achieving end-to-end fully automated stress detection. This has significant implications for stress prevention and intervention.

### 1.2 Related work

Using social media data for stress detection offers real-time insights, efficiency, and convenience, which are crucial for timely and effective interventions and counseling for users experiencing extreme stress. In this section, we review and summarize the techniques in stress detection among social media users, utilizing traditional machine learning and deep learning.

1. Stress Detection Based on Traditional Machine Learning

Before applying traditional machine learning for stress detection, it is essential to extract features from social media data. These features include linguistic, lexical and statistical, and domain-specific knowledge features. The most widely used method for extracting linguistic features is Linguistic Inquiry and Word Count (LIWC) [16], which performs quantitative analysis of word categories in textual content, such as positive or negative emotions [17]. Nguyen et al. demonstrated that LIWC performs well in depression classification [18]. Meyerhoff et al. used LIWC to study differences in communication patterns between depression patients and their intimate and non-intimate contacts [19].

Compared to linguistic feature extraction methods, lexical and statistical feature extraction methods such as Bag of Words (BOW), Term Frequency-Inverse Document Frequency (TF-IDF), and N-Gram, are more generalizable, as they statistically analyze the frequency and sequence of words in the text. According to Mustafa et al., TF-IDF improves the performance and accuracy of classifiers for identifying users with depression [20]. Mounika et al. found that combining TF-IDF with N-Grams enhances classifiers accuracy [21].

Domain-specific knowledge features mainly include topic and sentiment features. The extraction of topic features involves constructing word and sentence vectors to uncover stress-related latent semantics and topics. Common models include Non-negative Matrix Factorization (NMF), Latent Semantic Indexing (LSI), and Latent Dirichlet Allocation (LDA). Sentiment feature extraction mainly uses various sentiment-related lexical databases, such as Affective Norms for English Words (ANEW), NRC Emotion Lexicon, and Valence Aware Dictionary for Sentiment Reasoning (VADER). Zhang et al. demonstrated the effectiveness of the LDA model in identifying suicide-related topics [22]. Wolohan et al. and Tyshchenko et al. showed that combined feature extraction methods, such as BOW+LDA [23] and TF-IDF+LDA [24], improve accuracy. In sentiment feature extraction, Leiva et al. introduced positive, negative, and neutral sentiment features based on TF-IDF, demonstrating that methods incorporating sentiment features are more accurate than those using only TF-IDF [25].

In addition to feature selection and construction, the choice of algorithm is also crucial. Traditional machine learning-based stress detection algorithms include Logistic Regression (LR), Decision Tree (DT), Support Vector Machine (SVM), Naive Bayes (NB), and Random Forest (RF). Chiu et al. used LR to identify potential Major Depressive Episode and Severe Impairment (MDESI) in adolescents [26]. Islam et al. demonstrated that DT achieved superior results in detecting depression among Facebook users [27]. Yan et al. improved the identification rate of depression by using an ensemble SVM, which assigned lower weights to low-quality samples [28]. In Haque et al.’s study, RF was more effective and accurate in predicting depression among children and adolescents [29].

Overall, traditional machine learning have been widely applied to text classification tasks related to mental health, paving the way for detecting stress, depression, and suicide-related content on social media. However, to achieve more accurate results, these algorithms rely on manual feature engineering to extract user behavior features. This quantitative, word-count-based extraction method often overlooks contextual semantics and is prone to errors. Additionally, topic feature extraction methods face challenges such as the curse of dimensionality and difficulty in determining the number of topics. In summary, feature engineering requires extensive domain knowledge and expertise to identify relevant features for training, which consumes considerable time and effort from researchers, yet model performance often remains suboptimal.

2. Stress Detection Based on Deep Learning

Deep learning can automatically extract features from raw text vectors and has the ability to abstract and generalize concepts. It performs exceptionally well when handling massive amounts of social media data. In stress detection based on social media data, Convolutional Neural Networks (CNN), Long Short-Term Memory networks (LSTM), and Bidirectional Encoder Representations from Transformers (BERT) are the most popular and commonly used techniques [30]. Ckotsis et al. developed a classifier based on CNN and deep learning architectures to identify and categorize posts related to mental disorders on Reddit, achieving an accuracy of 91.08% [31]. Kim et al. applied CNN to identify users with potential mental health issues on Reddit and used the SMOTE algorithm to address data class imbalance, effectively improving the model’s accuracy [32].

CNN can automatically extract local information within text and possess strong parallel computing capabilities, yet it is unable to capture long-distance semantic information. In contrast, Recurrent Neural Network (RNN) variants such as Long Short-Term Memory (LSTM) and Gated Recurrent Unit (GRU) incorporate memory units that store contextual text information, addressing the issue of vanishing gradients in RNN models. This makes them advantageous for social media stress recognition. For instance, Amanat et al. constructed an emotion disorder prediction model based on LSTM-RNN, achieving better results than CNN [33]. Wani et al. applied LSTM, CNN, and hybrid models (CNN+LSTM) to depression signal datasets from various online social networks (OSNs), achieving accuracy rates of 99.02% for LSTM and 99.01% for CNN+LSTM models [34].

In recent years, the emergence of BERT [35] has significantly improved model performance in natural language processing (NLP). With BERT excelling in various NLP tasks, research based on the BERT for stress, depression detection, and suicide risk assessment has become mainstream. Boonyarat et al. introduced a BERT model integrated with linguistic features to predict suicidal intentions in Thai tweets, outperforming other baseline models [36]. EI-Ramly et al. were the first to use BERT to detect depression in Arabic social media posts, with the model greatly outperforming dictionary-based analysis and traditional machine learning [37]. Ilias proposed a novel method of infusing linguistic information into BERT to identify stress and depression in social media texts, achieving better performance than the original BERT model [38].

Overall, deep learning can automatically extract features, offering stronger robustness, generalization, and more precise detection performance. However, deep learning models have relatively large parameter sizes and often require large-scale data for optimal performance. On smaller datasets, their performance may not be as good as traditional machine learning. Consequently, scholars have begun adopting ensemble learning to combine the advantages of different models, achieving better performance than single models. For example, the BERT-BiLSTM model proposed by Zhang et al. integrates the advantages of BERT and BiLSTM, outperforming state-of-the-art benchmarks across all datasets [39]. Integrating BERT with other models through ensemble learning to maximize each model’s strengths has become a research trend in the field of stress detection [40,41].

### 1.3 Our work

Currently, there are no studies on stress detection models for postgraduates based on social media data and deep neural networks. The sources of stress for postgraduates differ significantly from those of other groups, making it impractical to directly apply models and conclusions from other studies to this population. Moreover, most current research focuses on English, with insufficient studies on resource-limited languages. This study aims to explore the potential of Chinese social media in detecting stress among postgraduates. Based on a hybrid BERT model, we developed an automated, real-time, and non-intrusive algorithm to detect the mental health, challenges, and stress of postgraduates.

This paper addresses several challenges and makes significant contributions:

1. The vast and complex data on social media platforms pose a significant challenge for automatic data acquisition and annotation. To address this, we developed a psychological stress database sourced from social media and utilized regular expressions, such as "I am pursuing a Ph.D./Master’s degree," "my advisor," and "my research group," to identify relevant social media posts. If a post met one of these matching criteria, we labeled the author as a postgraduate. Additionally, we manually tested a portion of the data to verify the accuracy of our identification method. This is the first real-time, automated, and non-intrusive stress detection system for postgraduates based on social media.
2. Current research on stress features extraction primarily relies on qualitative analysis. However, methods based on topic clustering are constrained by traditional Bag-of-Words models, which may fail to effectively incorporate textual semantics and contextual information. As a result, effectively extracting stress features from high-dimensional and sparse social media data remains a significant challenge. To address these issues, we propose a stress feature extraction algorithm based on an improved BERT-LDA framework. This algorithm utilizes the BERT model to capture character, word, and sentence-level information and their relationships, then integrates the LDA model to reduce dimensionality during the feature extraction process.
3. Modeling stress detection for postgraduates is more challenging compared to discrete data, as it requires consideration of group-level stress features and individual differences. To address this issue, we propose a named entity recognition model for stress detection based on the BERT-BiLSTM-CRF, which combines group-level stress features and user-level stress profiles. This approach achieves a fine-grained representation of stress features through multi-dimensional feature fusion, enabling real-time detection of stress levels and emotional health status across different groups.

## 2. Stress feature extraction among postgraduate students using social media data

### 2.1 Data acquisition and processing

We collected posts from three major Chinese social platforms, Douban, Zhihu, and Sina Weibo. We formulated corresponding data pre-processing strategies based on the types of data obtained from different platforms. To identify postgraduate students among social media users, we searched for keywords such as "doctoral student," "Ph.D. candidate," and "master candidate" in users’ public profiles. Users matching any of these keywords were labeled as postgraduate students. When user information was incomplete or missing, we used regular expressions to search for keywords such as "I am master/doctor candidate," "my advisor," or "my research group" in the post content. If the matching was successful, the poster was labeled as a postgraduate student. We collected social media data from May 2018 to May 2023, spanning the past five years. Table 1 shows the results of the original data acquisition and pre-processing. Ultimately, we obtained a total of 966,746 valid data points.

**Table 1:** Social media data acquisition and processing results.

Data Sources	Douban	Zhihu	Sina Weibo
**Search Term**	985, 211, Postgraduate, Ph.D., Master	Postgraduate, Ph.D., Master	Postgraduate, Ph.D., Master, Professor, Advisor
**Source**	Discussion Group	Topic	Topic
**Raw Data**	1206430	76951	47402
**Valid Data**	853390	68711	44645

### 2.2 An improved BERT-LDA algorithm for stress feature extraction

Latent Dirichlet Allocation (LDA) is a widely used document-topic extraction model consisting of a three-layer Bayesian probabilistic structure: the document layer, topic layer, and word layer. The basic idea is to synthesize a corpus from text data, select keywords from a set of topics within the text corpus, and then observe the distribution of these topics within the documents and the topic to which each word belongs. This process establishes relationships between document topics and words, generating topic vectors [42]. However, LDA is a traditional bag-of words model, it struggles to effectively integrate textual semantics and contextual information. To address this issue, we introduce Bidirectional Encoder Representation from Transformers (BERT) as an advanced pre-trained model. BERT falls under transfer learning, where the model is initially trained on a large corpus unrelated to the target task and then fine-tuned on the target domain’s set to complete specific NLP tasks. During BERT’s pre-training, two unsupervised prediction tasks are used: the Masked Language Model (MLM) and Next Sentence Prediction (NSP). These tasks enable BERT to perform self-supervised learning of textual context, allowing the model to comprehensively represent semantic relationships and syntactic structure in complex contexts. BERT learns relationship features at the character, word, sentence, and inter-sentence levels, demonstrating strong generalization capabilities [35].

**Fig 1:**
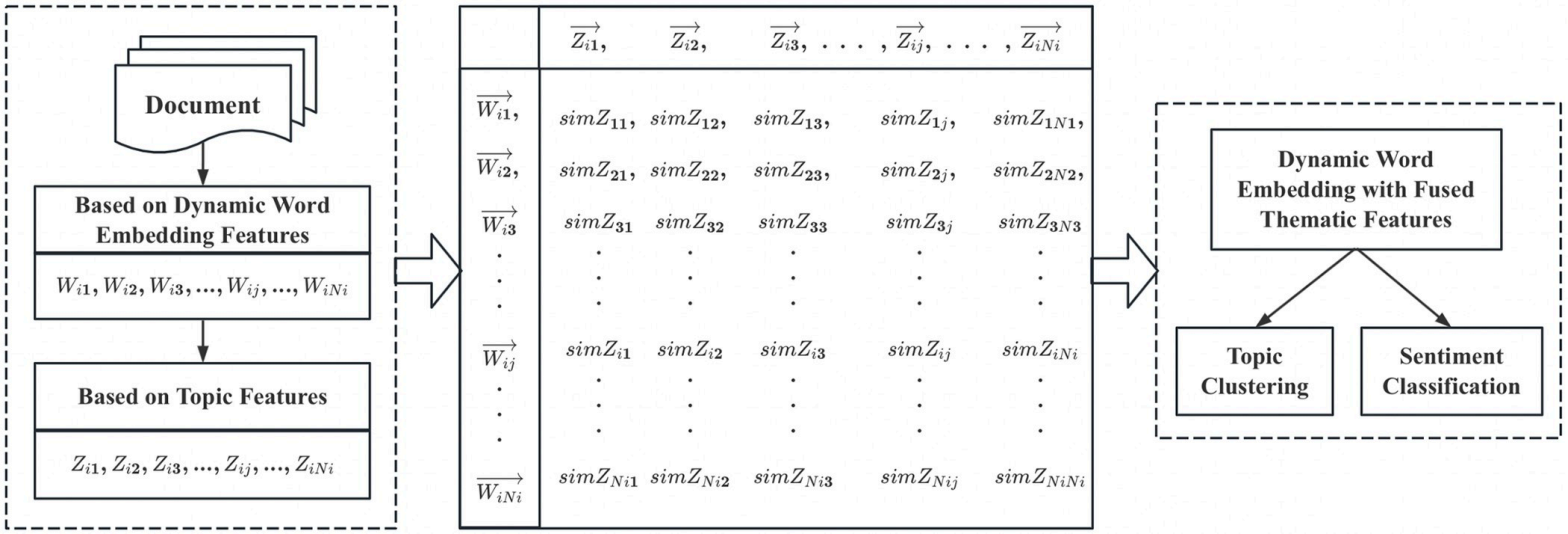
Stress feature extraction model based on BERT-LDA.

In this section, we introduce a novel algorithm for extracting stress features from large-scale complex texts using an improved BERT-LDA approach. Specifically, we have formulated new pre-training tasks for the BERT-LDA model. In addition to the original Chinese Wikipedia corpus, we incorporated sentiment and stress corpora from Sina Weibo and Baidu Tieba to enable the model to learn more about sentiment and stress information. We also introduced a publicly available annotated Sina Weibo dataset and a small number of stress-related sentiment annotation sets as deep pre-training corpora for the BERT model. This improved algorithm allows the model to fully integrate contextual semantic information, addressing the limitations of the LDA bag-of-words model. It trains more optimal topic vectors and deeply fuses the optimized topic vectors with BERT word vectors, resulting in more fine-grained and accurate stress feature extraction outcomes [43]. The proposed model framework is illustrated in Fig 1.

Fig 1 illustrates a high-dimensional semantic space comprising text sets, topics, and words. The steps of the stress feature extraction algorithm are as follows:

Step 1: Define that the document set $D=\{d_{i}|i\in \{1,2,\ldots M\}\}$ is composed of M documents d_i_. Among them, document $d_{i}=\{d_{is}|s\in \{1,2,\ldots S\}\}$ is composed of S sentences d_is_ and document $d_{i}=\{w_{ij}|j\in \{1,2,\ldots N_{i}\}\}$ is composed of *N*_*i*_ words *w*_*ij*_. We pre-process the data of document set D, such as word segmentation and stop words.

Step 2: Input the document output in step 1 into the BERT model for pre-training. Document d_i_ gets the dynamic word embedding $\left\{\vec{w_{i1}},\;\vec{w_{i2}},\ldots ,\;\vec{w_{ij}},\ldots ,\;\vec{w_{iN_{i}}}\right\}$, including word, sentence and position embedding. Add all the dynamic word embedding of document d_i_ and divide them by the total number of words N_i_ to get the document embedding $\vec{d_{i}}$, as shown in Eq (1):

$$\vec{d_{i}}=\frac{\sum_{j=1}^{N_{i}}\vec{w_{ij}}}{N_{i}}$$ (1)

Step 3: Input the dynamic word embedding $\left\{\vec{w_{i1}},\;\vec{w_{i2}},\ldots ,\;\vec{w_{ij}},\ldots ,\;\vec{w_{iN_{i}}}\right\}$ output from step 2 training into the LDA model, train the potential topic vector $\left\{\vec{z_{i1}},\;\vec{z_{i2}},\ldots ,\;\vec{z_{ij}},\ldots ,\;\vec{z_{iN_{i}}}\right\}$ of document d_i_, and conduct the inner product with the Dirichlet distribution θ_i_ of the topic. Finally, the optimal topic is obtained by calculating the minimum cosine distance between the potential topic vector and the document vector, as shown in Eqs (2) and (3):

$$\vec{z_{i}}=\sum_{j=1}^{N_{i}}\vec{z_{ij}}\theta_{ij}$$ (2)

$$sim(d_{i},z_{i})=\cos\left(\vec{d_{i}},\vec{z_{i}}\right)=\frac{\vec{d_{i}}∙\vec{z_{i}}}{\left|\left|\vec{d_{i}}|\times |\vec{z_{i}}\right|\right|}$$ (3)

Step 4: Add the potential topic vector obtained in step 3 and the word vector pre-trained by BERT in step 2 to obtain the dynamic word vector that integrates the topic features.

Step 5: Input the matrix obtained in step 4 into the BERT-LDA model to perform the task of topic clustering and sentiment classification.

### 2.3 Analysis of postgraduate students’ stress features based on social media

To extract and analyze the stress features of postgraduate students from social media data, we first pre-process the document set according to Step 1 of the algorithm in Section 2.2, which includes word segmentation. Next, we input the BERT pre-training model in step 2 for text vectorization to obtain word, sentence, and position embeddings. Then, we input the vectorized text into LDA for topic modelling, and select *α* = 50/*l*, *β* = 0.01 (*l* is the number of text topics) as a priori parameter combination to achieve the optimized topic distribution and minimum cosine distance. Finally, we embed the optimized topic vector into BERT-LDA to obtain dynamic word embeddings with topic features.

We utilized the BERT-LDA model trained in Section 2.2 for sentiment classification by inputting 966,746 text data, resulting in 853,594 negative texts and 113,152 positive texts. Given that stress is primarily associated with negative emotions (Bae & Lee, 2012), we designated the 853,594 negative texts as the stress document set *DT* = {*dt*_*i*_|*i*∈{1,2,…*M*}} and subsequently fed them into BERT-LDA for topic modelling. We employed the Perplexity-Var algorithm to evaluate the model’s ability for topic extraction and generalization. After iterative calculation, we found that when i = 20, the number of text topics reached the optimum. As indicated by previous research [43], the improved BERT-LDA algorithm for stress feature extraction enabled the extraction of topic information with dynamic word vectors, addressing the shortcomings of the LDA word bag model and enhancing the model’s topic granularity for text stress feature extraction. Additionally, it facilitated the provision of semantic information for topic dimensions in large-scale, complex text sentiment analysis tasks, thereby enhancing the accuracy of topic feature extraction.

Subsequently, we combined the subject IDs of similar features and extracted the high-frequency topic words of each feature based on their probability distribution. Referring to the classification outcomes in the literature [6–9] regarding stress features, we obtained the distribution of topic words for postgraduate students’ stress across five different features, as shown in Table 2. Topics 1, 6, 11, and 12 exhibited similar features and were therefore labeled as research stress based on the probability distribution of high-frequency topic words. Similarly, topics 0, 2, 14, and 18 were combined and designated as employment stress. Topics 3, 7, 8, and 9 were merged and labeled as emotional stress, while topics 4, 13, 15, and 17 were categorized as financial stress. Notably, topics 5, 10, 16, and 19 lacked significant stress features and were designated as other stress.

**Table 2:** Topic-Word" distribution of stress features of postgraduate students (the probability distribution of words in parentheses).

Topic ID	Stress features	High frequency topic words
1,6,11,12,	Research stress	Advisor (0.0479), Graduation (0.0219), Thesis (0.0195), Dropout (0.0175), Extension of graduation (0.0162), Boss (0.0143), Experiment (0.0106), Dismissal (0.0092), Scientific Research (0.0079), Suicide (0.0071)
0,2,14,18	Employment stress	On-the-job (0.0389), Work (0.0136), Discrimination (0.0129), Non-full-time (0.0097), Internship (0.0092), Major (0.0089), Organization (0.0088), Graduates (0.0088), Autumn recruitment (0.0082), Society (0.0074)
3,7,8,9	Affection stress	Unavailable (0.0296), Depression (0.02), Single (0.0127), Good-for-nothing (0.0061), Parents (0.0056), Affection (0.0047), Separation (0.0038), Spouse selection (0.0038), Marriage (0.0037), Boyfriend (0.0035)
4,13,15,17	Financial stress	Subsidy (0.0271), Fund (0.0172), Income (0.0157), Poor family (0.0153), Salary (0.0149), House purchase (0.0143), Monthly salary (0.0103), Involution (0.0099), Renumeration (0.0069), Beijing (0.0067)
5,10,16,19	Other stress	Fall (0.0169), Medical (0.0157), Postgraduate recommendation (0.0154), 985 (0.0151), Female doctor (0.0151), Retest (0.0146), Postdoctoral (0.0145), Evaluation (0.0144), Life (0.0140), Confusion (0.0139)

Feature 1 is research stress, which originates from multiple sources including interactions with advisor, graduation requirements, and thesis expectations. The relationship between students and advisors is the main factor causing this stress. The primary cause of this stress is the lack of effective supervision and accountability mechanisms between advisors and students. This issue leads to imbalances in various areas, such as production and labor within the economy, rights and obligations in law, the relationship between understanding and practice in philosophy, and the harmony between generations and orders in ethics.

Feature 2 is employment stress, which manifests in employer discrimination against non-top-tier universities and part-time postgraduate students. The root cause is the continuous expansion of postgraduate students, which contrasts with the limited availability of job-opportunities.

Feature 3 is affection stress, specifically the stress of marriage and love. Unmarried postgraduate students are often affected by social circles, age, and other factors, leading to increased stress in mate selection and marriage. Married postgraduate students face stress from family responsibilities and obligations.

Feature 4 pertains to the financial stress experienced by postgraduate students who are not fully financially independent, primarily due to high tuition fees and low subsidies. These factors contribute to a significant financial burden.

Feature 5 includes other stress, such as the stress of advancing to higher education, pressures faced by medical students, female doctors, and post-doctors researchers, as well as peer and interpersonal stress.

## 3. A multi-dimensional profile of stress among postgraduate students based on social media

By building a BERT-BiLSTM-CRF model to identify named entities in stress-related text, we can explore how factors such as gender, education background, and institution impact the stress levels of postgraduate students. This model provides a multi-dimensional portrayal of postgraduate stress and offers valuable theoretical insights that can assist relevant departments in proposing differentiated interventions based on the unique stressors experienced by postgraduates.

### 3.1 Stress text NER algorithm based on BERT-BiLSTM-CRF

**Fig 2:**
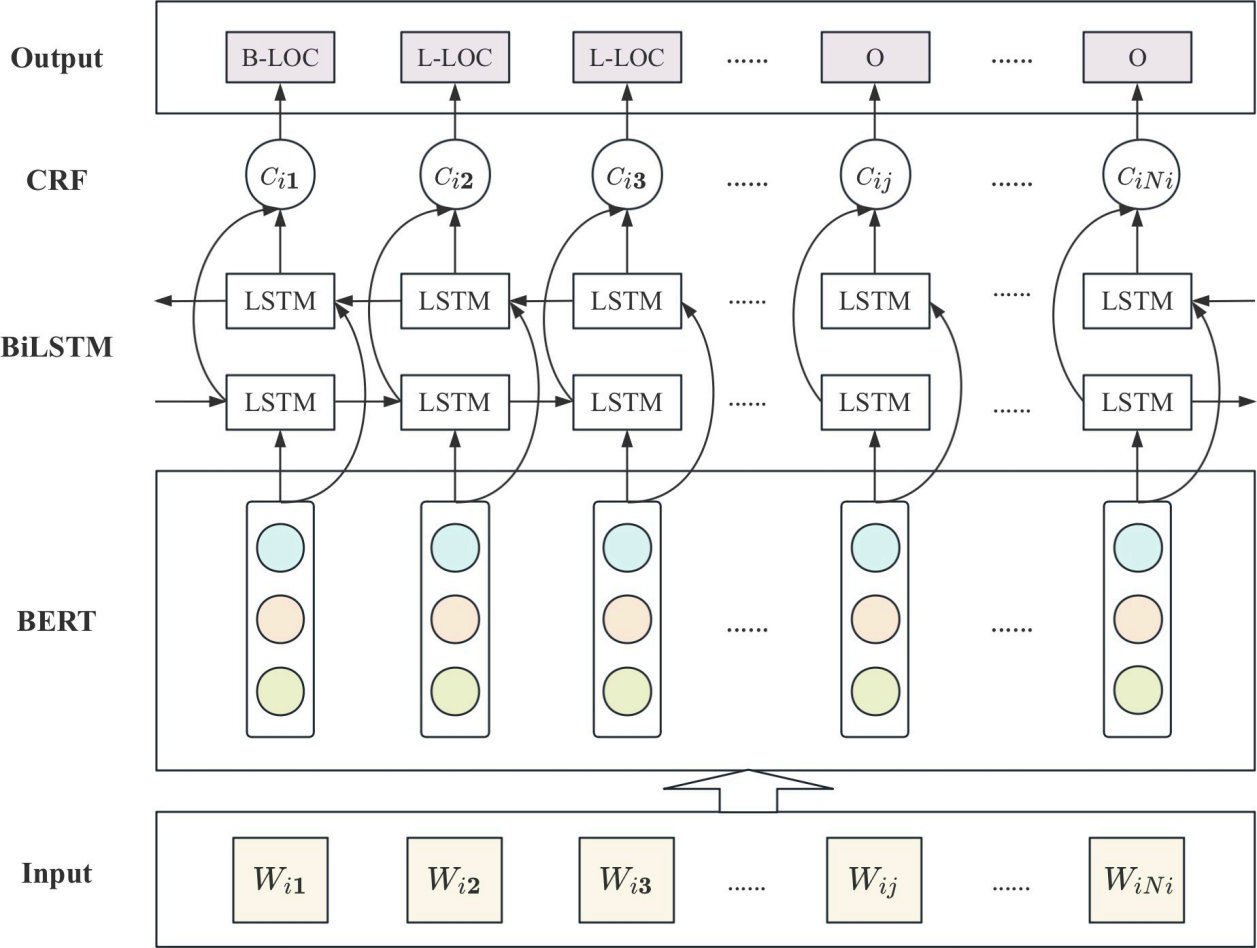
Model of Stress Text NER Based on BERT-BiLSTM-CRF.

Named Entity Recognition (NER) involves identifying and extracting specific types of entities from large volumes of unstructured text data, such as names of people, places, organizations, and other entities. It is one of the fundamental tasks in NLP. With the development of BERT, scholars have applied BERT to NER, achieving remarkable results [44–46]. Bidirectional Long Short-Term Memory (BiLSTM) is a type of RNN architecture consisting of two LSTM layers: a forward LSTM layer and a backward LSTM layer [47]. By combining information from both directions, the model can better capture contextual sequence information and long-distance dependencies between words in a sentence. The memory units and gating mechanisms of BiLSTM help alleviate the vanishing or exploding gradient problems that can occur in traditional RNNs. However, the BiLSTM model cannot handle dependencies between adjacent labels, and the addition of an overlapping CRF layer can compensate for the shortcoming. CRF is a sequence labeling model that considers the relationships between adjacent labels and performs joint probability analysis on the label sequence to provide the globally optimal label sequence, ensuring the rationality of the final predicted lables [48]. The stress text NER model consists of an input layer, a BERT feature representation layer, a BiLSTM text encoding layer, a CRF label decoding layer, and an output layer. The overall structure is shown in Fig 2.

The steps of the BERT-BiLSTM-CRF-based stress text NER algorithm can be divided into:

Step 1: Pre-process the stress dataset $DT=\{{dt}_{i}|i\in \{1,2,\ldots M\}\}$ according to part-of-speech tagging and named entity tagging, and input it into the BERT layer to obtain the text feature representation ${dt}_{is}=\{\vec{w_{i1}},\;\vec{w_{i2}},\ldots ,\;\vec{w_{ij}},\ldots ,\;\vec{w_{iN_{i}}}\}$, and then input it into the BiLSTM layer.

Step 2: For each word vector $\vec{w_{ij}}$, calculate its current value *h*_*t*_ in the LSTM hidden layer, as shown in Eq (4):

$$i_{t}=\sigma (\omega_{xi}x_{t}+\omega_{hi}h_{t-1}+\omega_{ci}c_{t-1}+b_{i})$$

$$f_{t}=\sigma (\omega_{xf}x_{t}+\omega_{hf}h_{t-1}+\omega_{cf}c_{t-1}+b_{f})$$

$$o_{t}=\sigma (\omega_{xo}x_{t}+\omega_{ho}h_{t-1}+\omega_{co}c_{t}+b_{o})$$

$$c_{t}=f_{t}{⊙c}_{t-1}+i_{t}⊙tanh(\omega_{hc}h_{t-1}+\omega_{xc}x_{t}+b_{c})$$

$$h_{t}=o_{t}⊙tanh(c_{t})$$ (4)

Where *i*_*t*_, *f*_*t*_ and *o*_*t*_ are the outputs of the input gate, forget gate and output gate, respectively, *c*_*t*_ is the value of the memory unit at time *t*, and *h*_*t*_ is the value of the hidden layer at time *t*, that is, the output of the entire LSTM layer at time *t*. *σ* is the activation function, *ω* is the weight matrix, and *b* is the offset vector. The forward and backward hidden vectors are concatenated element-wise to obtain the hidden layer output value *h*_*t*_ as shown in Eq (5).


$$h_{t}=[\vec{h_{t}},\overset{\leftarrow }{h_{t}}]$$ (5)

The embedded expression of the sentence is ${dt}_{is}=\{h_{i1},\;h_{i2},\ldots ,\;h_{ij},\ldots {,\;h}_{iN_{i}}\}$.

Step 3: Input ${dt}_{is}=\{h_{i1},\;h_{i2},\ldots ,\;h_{ij},\ldots {,\;h}_{iN_{i}}\}$ into CRF layer, set its corresponding output label as $y_{is}=\{y_{i1},\;y_{i2},\ldots ,\;y_{ij},\ldots {,\;y}_{iNi}\}$, and let B be the transfer matrix. Matrix element $B_{y_{ij},y_{ij+1}}$ represents the probability of transferring from label *y*_*ij*_ to label *y*_*ij*+1_, and k is the number of labels, then $B\in R^{\left(k+2)*(k+2\right)}$. Given that the sentence length is *N*_*i*_ characters, the score matrix of the output layer is $p={\left(p_{ij}\right)}_{N_{i}\times k}$, where $P\in R^{N_{i}*k}$, matrix element *p*_*ij*_, *y*_*ij*_ represents the probability that the *j-th* character of the *i-th* document is marked as *y*_*ij*_. Therefore, the total score function of the output tag sequence y_is_ is shown in Eq (6):

$$score(d_{is},y_{is})=\sum_{j=0}^{N_{i}}B_{y_{ij},y_{ij+1}}+\sum_{j=1}^{N_{i}}p_{ij,}y_{ij}$$ (6)

Finally, the Viterbi algorithm [49] is used to select the label sequence with the highest score as the final output of the model.

The proposed approach for NER of stress text is based on the BERT-BiLSTM-CRF architecture. Specifically, we utilize the stress text features output by BERT as input to the BiLSTM model, which allows us to extract contextual information. This information is then fed into the CRF layer for decoding, resulting in predictive annotation sequences. The model is then able to recognize and extract entities within the sequence. This methodology not only effectively captures text feature information but also improves the accuracy of entity recognition by enhancing the ability of global prediction tags.

### 3.2 Social media based multi-dimensional stress profile analysis of postgraduate students

To conduct a comprehensive multi-dimensional analysis of stress profile among postgraduate students based on social media, we utilized the BERT-BiLSTM-CRF stress text NER algorithm as outlined in Section 3.1. This algorithm facilitates the analysis and evaluation of various types of stress in social media posts, providing a nuanced understanding of the psychological and emotional states of postgraduate students.

We initially introduce the MSRA-NER named entity annotation set, along with the People’s Daily and Weibo annotation datasets, all provided by Microsoft Research Asia, and defined them as the training set DT_T. As these three public datasets contain ORG tags, we retained them while replacing other irrelevant tags with "O" to indicate that they do not correspond to any entity tag. Following Step 1 of the algorithm outlined in section 3.1, a portion of the text set DT, denoted as DT′, was manually annotated using the standard BIO annotation method. We used three distinct group feature differences of postgraduate students, namely SEX, EDU, and ORG, as labels for NER. These annotated data were then integrated into the training set DT_T. Details of the dataset are presented in Table 3.

**Table 3:** Data set statistics.

Data Set	Training Set *DT*_*T*	Testing Set	Label
**MSRA-NER**	46,364	4,365	ORG
**People’s Daily**	17,573	1,718	ORG
**Weibo**	1,350	270	ORG
**Annotation Set *DT*′**	1,331	1,000	SEX、EDU、ORG
**Total**	66,618	7,353	

Secondly, we input the three public available annotation datasets, which exclusively contain ORG tags, into the BERT layer for training and testing, following Steps 2 and 3 outlined in Section 3.1. We then input the annotation dataset, which includes SEX, EDU, and ORG tags, into the trained model and test it on the dataset’s test set after secondary training.

For training, we employed the Adam optimizer with a learning rate of 2e-5, and LSTM_Dim configured to 200. The batch_size for MSRA-NER and People’s Daily datasets was set to 128, while for the microblog dataset, it was set to 64. We also set the max_seq_len to 256 and the dropout rate to 0.5. To assess model performance, we computed precision, recall, and F_1_-score on the test set and compared the effectiveness of BiLSTM, BiLSTM-CRF, BERT-BiLSTM, and BERT-CRF models. The results are presented in Table 4.

**Table 4:** Overall comparison of effects of named entity recognition models.

Model	P(%)	R(%)	*F*_1_(%)
**BiLSTM**	87.64	86.18	86.53
**BiLSTM-CRF**	89.05	88.32	89.17
**BERT-BiLSTM**	91.16	90.75	90.87
**BERT-CRF**	91.03	91.42	91.98
**BERT-BiLSTM-CRF**	92.55	93.47	92.18

According to the results shown in Table 4:

1. BiLSTM is a mainstream neural network model, achieving a baseline performance of 86.53% without incorporating other components. However, compared to other models, BiLSTM lacks a well-defined input representation layer and text decoding layer, resulting in the lowest levels of precision, recall, and F1-score.
2. BiLSTM-CRF is a classic architecture for sequence labeling tasks. By introducing the CRF layer, the model alleviates the strong dependency relationships between sequence labels to some extent, achieving an F1-score of 89.17%, which is an improvement of 2.64% compared to the BiLSTM model.
3. BERT-BiLSTM is a BiLSTM model combined with a pre-trained language model, showing significant performance improvements due to dynamically vectorized input. However, experimental results indicate that the strong dependency issue between labels has not been fully resolved.
4. BERT-CRF is an effective applies the pre-trained language model to sequence labeling tasks. With the addition of the CRF layer, the model achieves competitive F1-scores.
5. BERT-BiLSTM-CRF architecture combines the pre-trained language model with the BiLSTM-CRF framework, resulting in a 3.01% improvement in F1-score compared to the traditional BiLSTM-CRF model. This improvement is due to the BERT model’s ability to dynamically generate contextual semantic representations of characters through its bidirectional transformer architecture. Compared to traditional word embedding methods, BERT provides a more precise understanding of semantic information and offers stronger feature extraction capabilities, validating the effectiveness of large language models.

Subsequently, we employed the BERT-BiLSTM-CRF model to evaluate the precision, recall, and F_1_-score of SEX, EDU and ORG entities, respectively. Table 5 presents the experimental results.

**Table 5:** Test results of test set on the model.

Label	P (%)	R (%)	*F*_1_(%)
**SEX**	95.6	96.46	95.99
**EDU**	96.64	93.02	94.56
**ORG**	88.47	90.86	89.31

**Fig 3:**
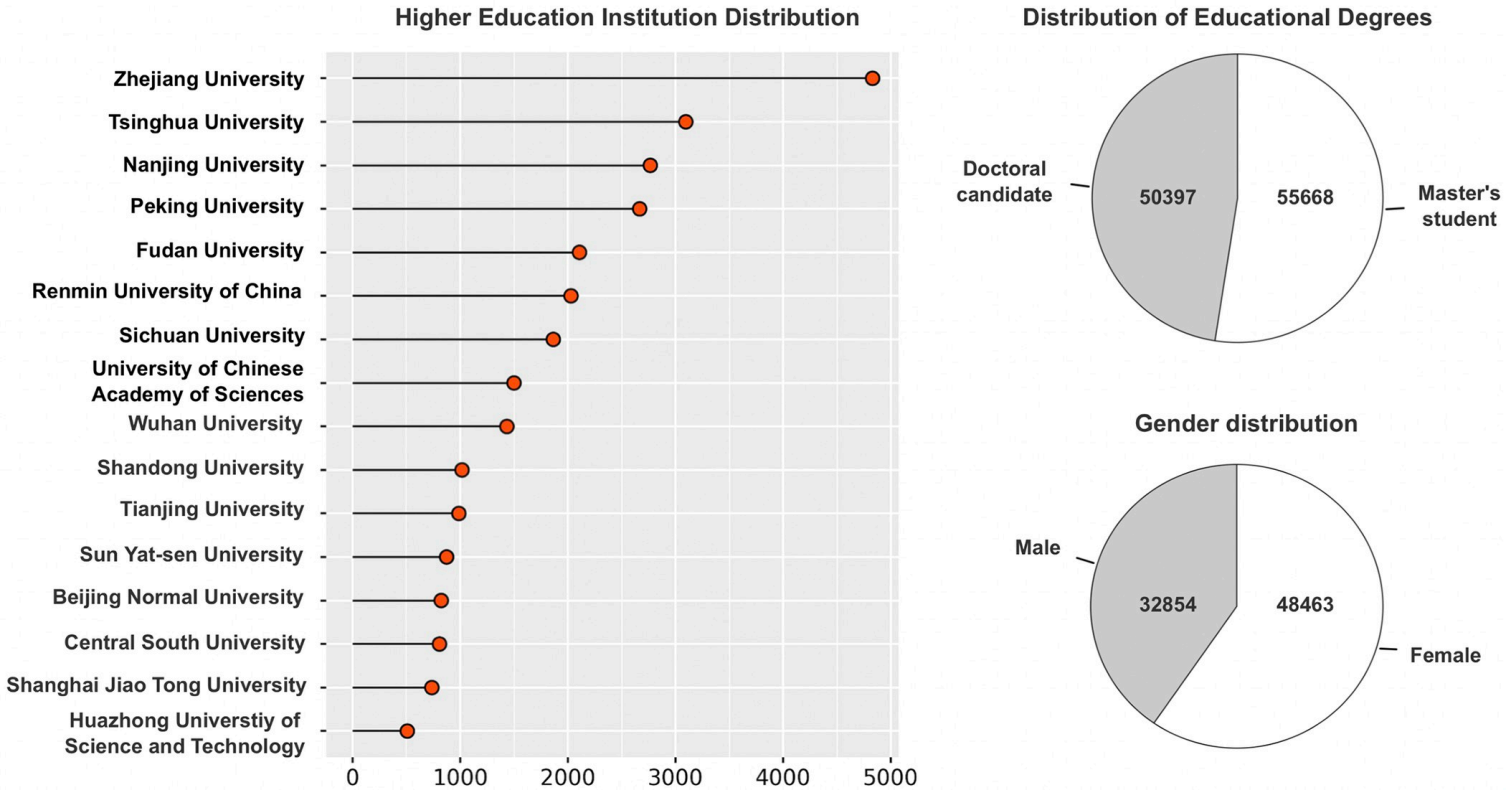
Multidimensional stress profile of postgraduate students.

The BERT-BiLSTM-CRF model, exhibiting superior performance after the second training, was utilized to identify and visualize entity names of universities (ORG), genders (SEX), and educational degrees (EDU) within the text set. The resulting output constitutes a multi-dimensional stress profile of postgraduate students, as demonstrated in Fig 3.

In Fig 3, we list the top 16 universities associated with stress. Notably, all listed universities are renowned worldwide. An analysis of the ORG tags extracted from the data reveals that world-class universities constitute 78.54%, while non-top-tier universities comprise the remaining 21.46%. However, the actual proportion of world-class universities in China is less than 5%. Therefore, it is reasonable to infer that postgraduate students enrolled in world-class universities may be more inclined to disclose their institution’s name on social media platforms, whereas those attending non-top-tier universities might be more prone to conceal it. Furthermore, the majority of master’s and doctoral degree programs in China are offered by prestigious universities, potentially leading to a higher frequency of relevant comments from students at such institutions. However, it is important to acknowledge that the algorithm employed in this study may not accurately identify all ORG tags due to the diverse expressions used on social media platforms. Additionally, users often obfuscate personal information, referring to their institution as "Top-number" or "non-top-tier" universities, which further complicates the identification of relevant ORG tags.

An analysis of the distribution of educational backgrounds among users revealed that the number of users pursuing a master’s degree was approximately equal to those pursuing a doctorate. However, in China, the average ratio of students enrolled in master’s programs to those in doctoral programs is approximately 4:1 [50]. This discrepancy suggests that individuals pursuing a doctorate may be more inclined to express their stress on social media compared to master’s students. This observation may reflect that the higher academic demands and associated stress often experienced by doctorate students relative to master’s students. Furthermore, the gender distribution of users on social media platforms shows a significant predominance of female users. The observation aligns with the tendency of female users to exhibit a higher propensity for expressing negative emotions and engaging in self-disclosure on social media compared to male users [51].

## 4. Fine-grained representation and intervention strategy of stress level within multi-dimensional feature fusion

The objective of this study is to conduct a comprehensive analysis of fine-grained feature representation among postgraduate students, utilizing multi-dimensional feature fusion techniques. By examining various label features, we aim to gain an in-depth understanding of the stress experienced by postgraduate students. This analysis will facilitate the identification of patterns and relationships among features, enabling the development of targeted intervention and counselling strategies. Through this approach, we aim to contribute to the expanding research on stress and well-being among postgraduate students and provide practical recommendations for universities and mental health professionals.

### 4.1 Fine-grained representation of stress level

**Fig 4:**
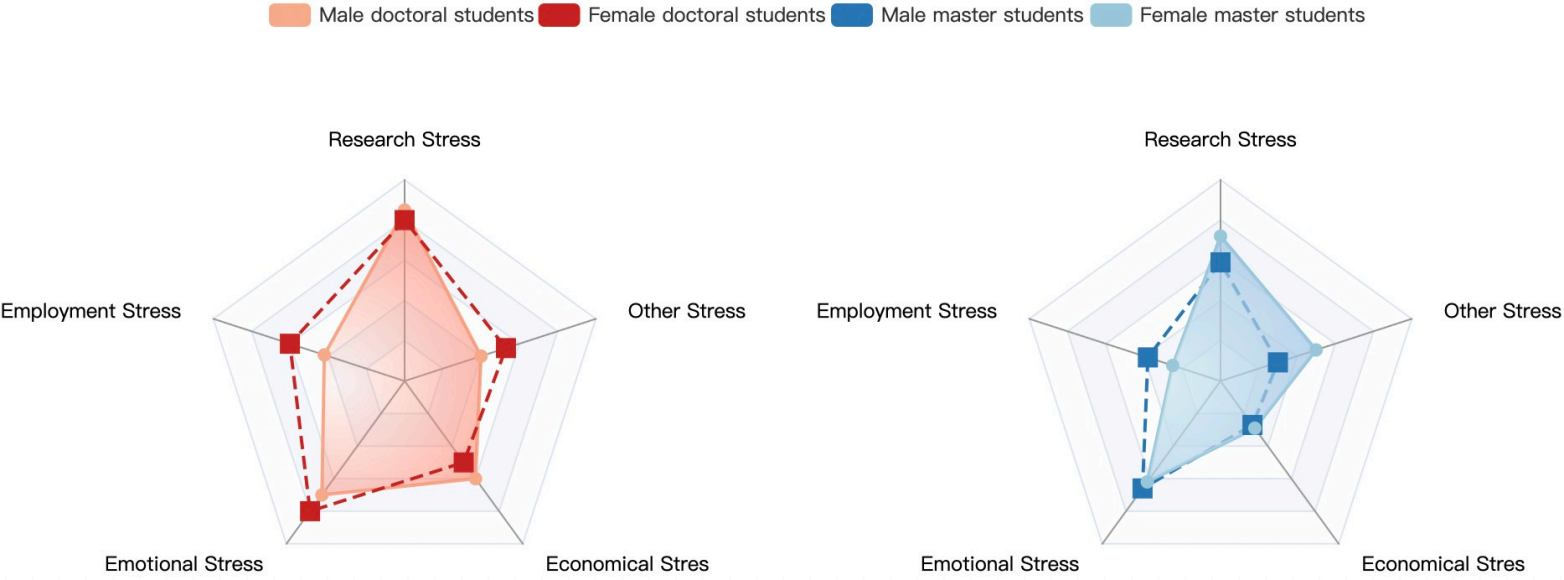
Fine-grained analysis of stress on the dimensions of SEX and EDU.

We extracted text containing both the SEX (gender) and EDU (education) labels using the results from the BERT-BiLSTM-CRF entity recognition algorithm described in Section 3.2. The extracted text was then categorized into four mixed-label categories: "male doctoral students," "female doctoral students," "male master’s students," and "female master’s students," with corresponding text counts of 14,831, 20,248, 12,640, and 24,059, respectively. By matching the four categories with the feature words generated by the BERT-LDA model in Section 2.3, we calculated the probabilities of the occurrence of five feature words in each categories. The distribution results are shown in Fig 4.

Based on the findings presented in Fig 4, research stress emerges as the primary stress factor for male doctoral students and female master’s students, while affection stress predominates for female doctoral students and male master’s students. Additionally, female postgraduate students experience higher stress levels compared to their male counterparts, and doctoral students generally experience higher levels of stress than master’s students. However, gender differences do not appear to be the primary determinant of stress levels among postgraduate students. The academic roles of postgraduate students, combined with their typically high levels of academic engagement and education, may mitigate the influence of gender on stress levels.

Furthermore, our analysis reveals that affection and research stress are the main contributors to the stress levels of postgraduate students. This contrasts with previous research findings [52–54], which often emphasize employment and financial stress as primary stressors for postgraduate students. Interestingly, postgraduate students who actively express and share their affection needs on social networks tend to experience lower levels of financial and employment stress. This suggests that traditional surveys methods or interviews may constrain individuals within this group due to prevailing stereotypes of postgraduate student identities and social expectations, leading them to suppress their affection needs and stress. In contrast, virtual social networks provide a more conducive environment for the expression of emotions.

**Fig 5:**
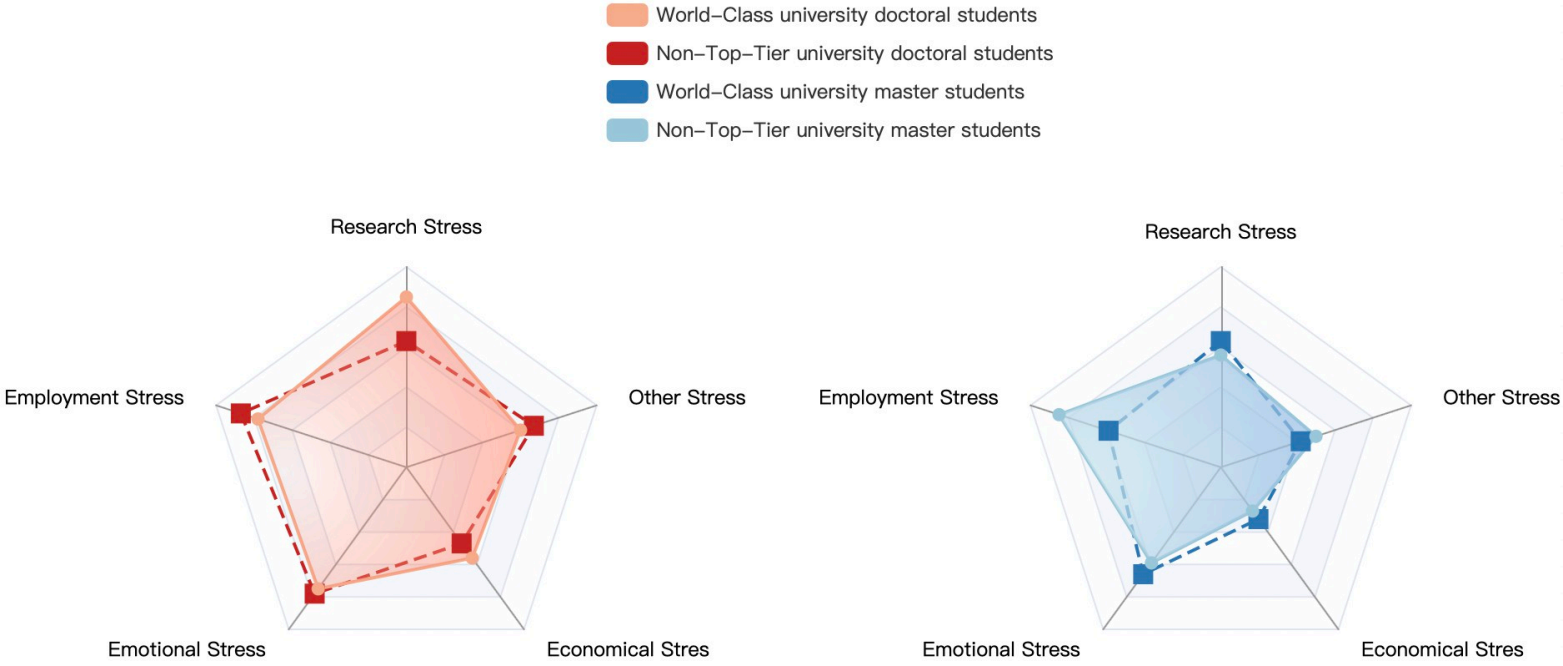
Fine-grained stress analysis on the dimensions of EDU and ORG.

Text containing the labels EDU (educational degrees) and ORG (universities) was extracted and matched with the feature words. The ORG label was further divided into "world-class universities" and "non-top-tier universities," resulting in four mixed-label text categories: "doctoral students from world-class universities," "doctoral students from non-top-tier universities," "master’s students from world-class universities," and "master’s students from non-top-tier universities." The corresponding text counts for these categories are 10,350, 7,652, 16,802, and 18,516, respectively. The distribution of the occurrence probabilities of the five feature words in these four mixed-label text categories is shown in Fig 5.

Fig 5 shows that master’s and doctoral students from non-top-tier universities tend to experience higher levels of research and employment stress compared to their counterparts from world-class universities. Furthermore, research, employment, and affection stress emerge as primary stressors among doctoral students overall. Notably, non-top-tier university master’s students exhibit significantly heightened stress levels, particularly regarding employment stress, which is their primary stressor. This observation contrasts with that of master’s and doctoral students from world-class universities, where doctoral students report heigher employment stress levels compared to master’s students.

The observed disparities between postgraduate students from world-class and non-top-tier universities can be attributed to differential access to educational resources, including infrastructure and research support. As a result, postgraduate students from world-class universities often possess a strong theoretical foundation and enhanced research capabilities, giving them comparative advantages in both research and employment pursuits. Conversely, postgraduate students from non-top-tier universities experience higher levels of employment stress, particularly among specific subgroups such as professional master’s, part-time master’s and doctoral students, in-service master’s and doctoral students, and those from non-top-tier universities, where instances of employment discrimination are more prevalent. These factors collectively contribute to the heightened stress levels among postgraduate students form non-top-tier universities compared to their counterparts from world-class universities.

### 4.2 Intervention and guidance strategies for stress

To summarize, the findings suggest that all eight types of postgraduate students experience substantial levels of research and emotional stress. Based on the analysis of postgraduate students’ stress features in Section 2.3, the following conclusions can be drawn:

1. The advisor-advisee relationship is the primary source of research stress among postgraduate students, highlighting the urgent need for reform within this traditional structure. Firstly, establishing a legal framework that clearly delineates the rights and obligations of both parties is essential, thereby enhancing the autonomy of the advisor’s guidance and the academic freedom of the postgraduate student. Secondly, innovative models for the advisor-advisee relationship, such as the dual-advisor system or the advisor group system, offer potential solutions for mitigating the academic authoritarianism inherent in conventional arrangements, while also providing postgraduate students with greater agency in selecting their advisors. Implementing these reforms can help postgraduate education create a more supportive and empowering environment that fosters creativity, innovation, and collaboration scholarship.
2. Universities should focus on the affection-related stress experienced by postgraduate students and implement measures to update outdated attitudes towards marriage, love, and educational paradigms. Firstly, it is necessary to establish a comprehensive curriculum and educational materials that address marriage and relationship education, respecting and acknowledging the romantic and relational needs of postgraduate students while fostering healthy attitudes towards these aspects. Secondly, it is crucial to develop a robust management framework catering to the marriage and relationship needs of postgraduate students. For instance, universities should provide legal protections for postgraduate students of legal marriage age, including provisions for marriage and maternity leave, housing, subsidies, and other relevant considerations. These measures should also extend to postgraduate students who are already married or pregnant.
3. The financial challenges faced by male doctoral students and those enrolled in top-tier universities necessitate a two-pronged approach. Firstly, there is a need to optimize funding policies for doctoral students by increasing the funding amount and extending the duration of financial assistance where necessary to ensure their basic living expenses are covered. Secondly, supervisors should enhance research funding support for doctoral candidates, which could involve increasing the number of research and teaching assistant positions and providing additional financial resources tailored to special groups such as married or parenting students and those from impoverished backgrounds. These initiatives are essential for alleviating the financial stress experienced by doctoral students, thereby enabling them to focus their energies on their research pursuits, leading to enhanced productivity and academic success.
4. To effectively address the employment stress faced by male master’s students and those enrolled in non-top-tier universities, particularly concerning employment discrimination against professional, part-time, on-the-job, and non-top-tier master’s programs, the following measures should be implemented: Firstly, a comprehensive improvement in the training system and quality is imperative. This involves allocating additional high-quality educational resources to non-top-tier universities to boost their social recognition and eliminate employment discrimination at its root. Secondly, there is a need to amplify the promotion and ensure the employment security of vocational education, aiming to improve the recognition and acceptance of these cohorts by employers. Thirdly, it is essential to establish a contemporary talent selection methodology while eliminating outdated concepts and prejudices. This ensures that individuals are assessed based on their skills, abilities, and knowledge, rather than discriminatory factors. By implementing these measures, the employment stress experienced by male master’s students and those in non-top-tier universities can be effectively mitigated, fostering a more equitable and inclusive employment environment.

## 5. Conclusion

This study proposes an improved BERT-LDA algorithm for stress features extraction and a BERT-BiLSTM-CRF algorithm for stress text NER. These advancements facilitate accurate analysis of stress features and the constrution of multidimensional stress profiles for postgraduate students, providing practical recommendations for targeted interventions and guidance to alleviate psychological stress. This is the first real-time, automated, and non-intrusive stress detection system for postgraduates based on social media, and the feasibility of deep learning-based models for stress detection and analysis among postgraduate students has been thoroughly validated.

Additionally, we leveraged the BERT pre-training model for stress text analysis and entity recognition, using BERT’s output as inputs for downstream tasks such as LDA and BiLSTM-CRF. This approach not only reduces the workload of downstream tasks but also achieves higher accuracy in stress feature extraction. We compared our proposed method with state-of-the-art techniques and other hybrid models using various performance metrics, and our model achieved an F1 score of 92.18%, outperforming the aforementioned methods.

In summary, the development of deep learning and NLP techniques has facilitated the investigation of stress and emotional health among postgraduate students using social media data. Nonetheless, this study has several limitations. Firstly, social media texts are mostly short, which limits the model’s ability to capture contextual information, making it difficult to detect irony and sarcasm in online texts and leading to subjectivity in interpretation. Secondly, the amount of time spent by postgraduate students on social media varies greatly [55], with users from central and coastal cities and economically developed areas being more representative [56]. Therefore, there may be a "digital divide" in the dataset used in this study, which only partially reveals the stress status of postgraduate students and does not represent the entire postgraduate population [57]. Finally, due to the limitations of social media platforms, it is impossible to access all posts from relevant users. However, factors such as the timing of posts, the time when stress was first detected, and the duration of stress can aid in early stress risk detection and prioritize cases that require immediate attention [58]. In the future research, we will enhance the ability of deep learning models to detect irony and sarcasm and attempt to integrate knowledge related to postgraduate stress into a knowledge graph, combined with deep learning methods, to construct interpretable stress detection models.

## Supporting information

S1 File (ZIP)

S2 File (ZIP)

S3 File (ZIP)

S4 File (ZIP)
